# Influence of cyclosporine A on glomerular growth and the effect of mizoribine and losartan on cyclosporine nephrotoxicity in young rats

**DOI:** 10.1038/srep22374

**Published:** 2016-03-07

**Authors:** Ji Hong Kim, Yeon Hee Lee, Beom Jin Lim, Hyeon Joo Jeong, Pyung Kil Kim, Jae Il Shin

**Affiliations:** 1 Department of Pediatrics, Gangnam Severance Hospital, Yonsei University College of Medicine, Seoul, Korea; 2 Department of Pediatrics, Yonsei University College of Medicine, Seoul, Korea; 3 Department of Pathology, Yonsei University College of Medicine, Seoul, Korea; 4 Department of Pediatric Nephrology, Severance Children’s Hospital, Yonsei University College of Medicine, Seoul, Korea

## Abstract

The aim of this study was to evaluate the influence of cyclosporine A (CsA) on glomerular growth and the effect of mizoribine (MZR) and losartan (LSAR) on CsA-induced nephropathy in young rats. Six-week-old male Sprague-Dawley rats maintained on a low salt diet were given CsA (15 mg/kg), CsA and LSRT (30 mg/kg/day), CsA and MZR (5 mg/kg), or a combination of CsA, LSRT, and MZR for 4 and 7 weeks (two experiments) and compared with control group (olive oil-treated). Histopathology and glomerular size, inflammatory and fibrotic factors were studied. The score of acute CsA toxicity significantly decreased in the CsA + MZR group compared to the CsA group (p < 0.01). MZR and MZR + LSRT reduced tubulointerstitial fibrosis and TGF-β1 mRNA expression at 7 weeks. Osteopontin (OPN) mRNA expression was decreased at 7 weeks in MZR + LSRT (p < 0.01). Glomerular area decreased CsA group and recovered in MZR (p < 0.01) and MZR + LSRT (p < 0.01) at 7weeks. This study demonstrated that MZR and LSRT had suppressive effects on inflammatory process in chronic CsA nephropathy and led to improvement of tubular damage, tubulointerstitial fibrosis and arteriolopathy by down regulation of OPN and TGF-β1 and glomerular size contraction.

Cyclosporine A (CsA) is a calcineurin inhibitor (CNI) which interferes with the calcineurin phosphatase, leading to inhibit the production of cytokines such as interleukin-2 needed for the activation of T cells^1^. Since its introduction in the 1980s in suppressing transplantation rejection, CsA brought a dramatic improvement in transplant outcomes in multiple organs^2^. In addition to inhibition of the action of T-cell-related cytokines, it can also stabilize the actin cytoskeleton of podocytes^3^.

However, CsA can cause acute and chronic CsA nephrotoxicity^4^,^5^. The biggest problem is chronic CsA nephrotoxicity after long-term use such as in transplant patients or steroid resistant nephrotic syndrome. The incidence of chronic CsA nephrotoxicity is about 30–40% in long-term use and the risk factors are reported to be steroid-resistant patients, the duration of CsA treatment, the duration of heavy proteinuria during CsA treatment (>30 days), an age younger than 5-years at the start of CsA and higher CsA trough or C2 (CsA level 2 hours after administration) drug levels^6^,^7^,^8^,^9^,^10^.

There have been some studies on the protective effects of various drugs such as mycophenolate mofetil, mizoribine and RAS blocekrs on chronic CsA nephrotoxicity in animals^11^,^12^,^13^. However, there has been no report on the effect of these drugs on acute or chronic CsA nephrotoxicity regarding glomerular growth in young rats. In addition, because a younger age at the start of CsA is one of the factor for the development of chronic CsA nephrotoxicity in children with nephrotic syndrome^8^, we thought that understanding of acute or chronic CsA nephrotoxicity in young animals might provide an insight on the pathophysiology of CsA nephrotoxicity in children.

The aim of this study was to evaluate the influence of cyclosporine A (CsA) on glomerular growth and the protective effect of concurrent administration of mizoribine (MZR) and losartan (LSAR) on CsA-induced acute and chronic nephropathy in young rats.

## Results

### Changes in body weight and blood chemistry results after 4-week administration of CsA

At the first experiment, in CsA/LSRT (100 mg/L) group, the proportion of young rats who survived 4 weeks after CsA administration was low and survived mice also did not eat well and the weight gain was blunted. The dose of LSRT was determined as 50 and 100 mg/L with the same dose to a similar age of rats from other experiments. We speculate that the dose of LSRT capacity (50 and 100 mg/L) was not tolerable in young rats administered in combination with CsA.

At 4 weeks after administration of CsA, there was no difference in the degree of increase in body weight between the CsA group and the control (olive oil as a vehicle) group. The CsA + LSRT 100mg/L group showed significantly decreased weight gain without statistical significance (see Supplementary Table S1). There was no significant difference in blood CsA concentration among the groups. Compared to the CsA group, serum BUN levels were significantly decreased in the CsA + MZR group (p < 0.01) and serum creatinine levels were significantly decreased in the CsA + MZR group and CsA + LSRT 50 mg/L + MZR group (p < 0.01) (see Supplementary Table S2).

### Histological renal toxicity after 4-week administration of CsA

**Figure 1:**
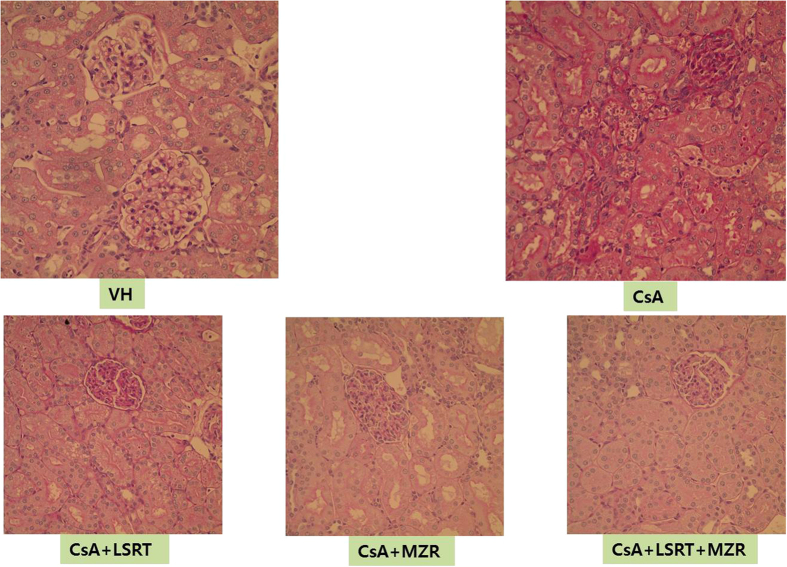
Acute tubular change in rat kidney due to CsA nephrotoxicity after 4 weeks. CsA-treated group showed significant increase of tubular epithelial vacuolar degeneration and drop out. Mizoribine-treated group showed significant lesser histologic change than CsA treated group (PAS stain, ×400).

After 4-week administration of CsA, the typical chronic CsA nephrotoxicity did not appear clearly and vacuolization and detachment from the tubular basement membrane were shown which are mainly the phenomenon of the acute tubular damage. The score of acute CsA toxicity was significantly higher in the CsA group compared to that in the control group (p < 0.01) and it significantly decreased in the CsA + MZR group compared to the CsA group (p < 0.01). However, the CsA + LSRT group did not show significant changes in the score compared to the CsA group (Table 1 and Fig. 1).

### Changes in glomerular area after 4-week administration of CsA

Glomerular area (Bowman’s and the glomerular tuft) significantly decreased in the CsA group than that in control group (VH group) (p < 0.01). Glomerular area was significantly increased in the CsA + LSRT (50mg/L), CsA + MZR, CsA + LSRT (50 mg/L) + MZR compared to that in the CsA group (Table 2). The weight of kidneys showed a decrease in all the groups compared to the control group (p < 0.05) (Table 2).

### Osteopontin (OPN, spp1) and TGF-β1 mRNA using quantitative real-time PCR and immunohistochemistry after 4-week administration of CsA

**Figure 2:**
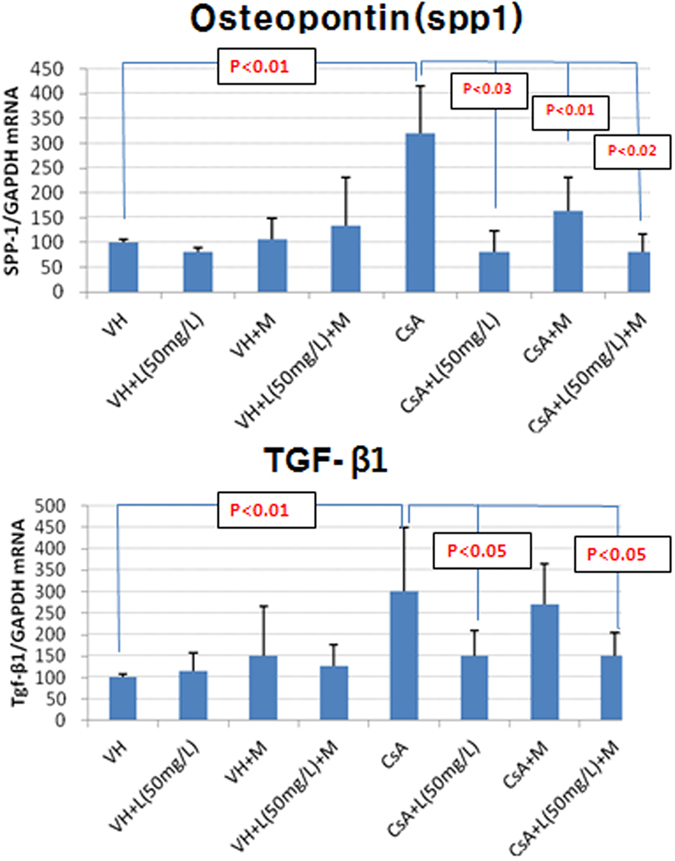
Expression of osteopontin (spp1) and TGF-β1 mRNA in rat kidney detected by real-time PCR. (Abbreviations: VH; vehicle (olive oil), M; mizoribine, L; losartan, CsA; cyclosporine A, spp1; Secreted Phosphoprotein 1, TGF; transforming growth factor, Data are expressed as mean ± SD).

Osteopontin (Secreted Phosphoprotein 1) OPN (spp1) mRNA expression in renal tissue using the quantitative real-time PCR was significantly higher in the CsA group compared with the control group (p < 0.01) and significantly lower in CsA + MZR group (p < 0.01), CsA + LSRT group (p < 0.03), CsA + MZR + LSRT group (p < 0.02) than in the CsA group. TGF-β1 mRNA expression in renal tissue was significantly higher in the CsA group compared with the control group (p < 0.01) and significantly lower in the CsA + LSRT group (p < 0.05) and CsA + MZR + LSRT group (p < 0.05) than in the CsA group. (see Supplementary Table S3, Fig. 2). Immunohistochemistry of osteopontin and TGF-β1 after 4-week administration of CsA is presented in Supplementary Figure S1.

### Changes in body weight and blood chemistry results after 7-week administration of CsA

According to prior experience of intolerability in the first experiment, we adjusted the dose of LSRT to 35 mg/L at the second experiment for chronic renal toxicity and this experiment showed that the growth state of the rats was good.

Body weight decreased significantly in the CsA group compared with the control group (p < 0.05) (see Supplementary Table S4). BUN levels were significantly increased in the CsA group (p < 0.05), while serum creatinine levels did not differ between the CsA group and the control group (see Supplementary Table S5).

### Histological chronic renal toxicity after 7-week administration of CsA

**Figure 3:**
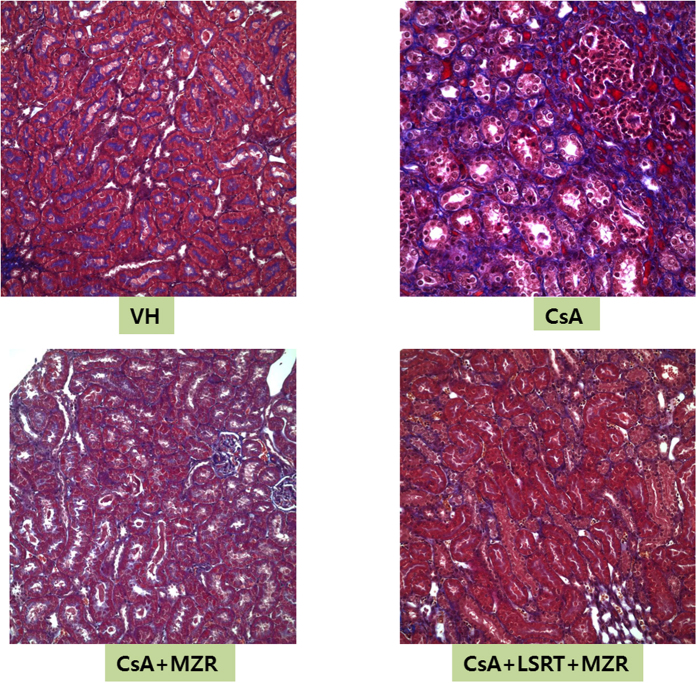
Histologic change due to chronic CsA nephrotoxicity after 7 weeks. CsA-treated group showed significant increase of interstitial fibrosis due to chronic CsA nephrotoxicity and mizoribine, mizoribine + losartan-treated group showed significant lesser interstitial fibrosis than CsA-treated group (trichrome stain, ×200).

When the typical histological findings in chronic CsA nephrotoxicity was scored after 7-week administration of CsA, the score of tubular atrophy, interstitial fibrosis and mononuclear cell infiltration was significantly higher in the CsA group compared to that in the control group (p < 0.01). There was no difference in tubular atrophy and mononuclear cell infiltration among the CsA-treated groups, but interstitial fibrosis was significantly lower in the CsA + MZR group (p < 0.01), and the CsA + LSRT + MZR group (p < 0.05) compared to the CsA alone group (Table 3 and Fig. 3). Arteriolar lesions were significantly higher in the CsA group compared with the control group (p < 0.01), but those decreased significantly in the CsA + LSRT + MZR group compared with the CsA alone group (p < 0.05) (Table 4 and Supplementary Figure S2).

### Changes in glomerular area after 7-week administration of CsA

Kidney weight decreased significantly in the CsA group (p < 0.01) than in the control (VH group) group, but there was no difference among CsA-treated groups. The glomerular bowman’s area, and the glomerular tuft area were significantly reduced in the CsA group compared with the control group (VH group) (p < 0.01), but those were significantly increased in CsA + MZR and CsA + LSRT + MZR groups (p < 0.01) (Table 5).

### Osteopontin (OPN, spp1) and TGF-β1 mRNA using quantitative real-time PCR and immunohistochemistry after 7-week administration of CsA

**Figure 4:**
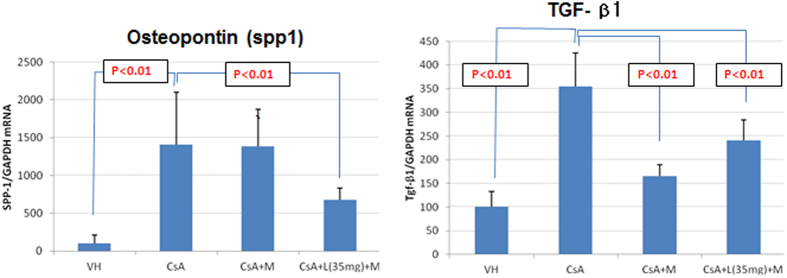
Expression of osteopontin (spp1) and TGF-β mRNA in rat kidney detected by real-time PCR. (Abbreviations: VH; vehicle (olive oil), M; mizoribine, L; losartan, CsA; cyclosporine A, spp1; Secreted Phosphoprotein 1, TGF; transforming growth factor, Data are expressed as mean ± SD).

After 7-week administration of CsA, renal OPN (spp1) and TGF-β1 mRNA expression in the CsA alone group increased by 1.4 and 3.5 times, respectively, compared to the control group (VH) (p < 0.01). Compared to the CsA alone group, renal OPN (spp1) mRNA expression was significantly reduced in the CsA + LSRT + MZR group (p < 0.05) and renal TGF-β1 mRNA expression was significantly reduced in the CsA + MZR and CsA + LSRT + MZR group (p < 0.01) (see Supplementary Table S6 and Fig. 4).

Immunohistochemistry of osteopontin and TGF-β1 after 7-week administration of CsA is presented in Supplementary Figure S3.

### Changes in anti-rat ED-1 positive cells CsA after 7-week administration of CsA

After 7-week administration of CsA, ED-1 positive cells were significantly increased in the CsA-treated groups than in the control (VH) (p < 0.01) compared with the CsA alone group on the immunohistochemistry and those were significantly decreased the CsA + MZR and the CsA + LSRT + MZR groups (p < 0.01) (see Supplementary Table S7, Supplementary Figure S4).

## Discussion

CsA nephrotoxicity is divided into acute and chronic forms. Acute CsA nephrotoxicity occurs through acute reversible vascular contraction by direct toxicity of the renal vessels and a decrease in glomerular filtration rate by systemic hemodynamic changes to CsA treatment^14^,^15^ and renal function can be normalized if the use of CsA is stopped. The characteristic histological changes may represent vacuolization and detachment of the tubular cells and activation of the sympathetic nervous system and the renin-angiotensin system (RAS), endothelin (ED)-1, nitric oxide has been known to be involved in acute CsA nephrotoxicity^16^,^17^,^18^,^19^.

The pathogenic mechanism of chronic CsA nephrotoxicity is not clear, but appears to be involved in direct tubular injury or vascular injury and chronic ischemia in kidneys, leading to tubular atrophy and interstitial fibrosis^4^,^5^,^20^,^21^. If the damage of renal tubular cells and interstitium is caused by CsA, renal tubular cells are mainly eliminated by apoptosis^22^,^23^ and renal injury progresses through hyperactivation of the immune mediators such as angiotensin II, transforming growth factor (TGF)-β1, osteopontin (OPN) and macrophages in addition to direct toxic effects of CsA^24^,^25^.

ED-1 has a strong vasoconstrictor action and is involved in the renal blood flow, glomerular filtration rate, reabsorption of sodium by controlling vascular tone^18^. It can also affect the glomerular histological changes through accumulation of the extracellular matrix and interstitial fibrosis^18^. OPN is primarily expressed in the distal tubule and Henle’s loop of the renal medulla and is not expressed normally in the renal cortex^17^,^24^. CsA administration can cause macrophage infiltration and interstitial fibrosis by increasing the expression of OPN in the kidneys^17^,^24^. TGF-β1 is known to be an important cytokine in the pathogenesis of glomerulosclerosis and tubulointerstitial fibrosis^25^ and expression of TGF-β1 increased in proportion to the CsA dose. An increase of TGF-β1 by CsA is connected to the tubulointerstitial fibrosis through an increase in extracellular matrix and RAS activation^26^,^27^.

The prevention of chronic CsA nephrotoxicity is to stop or reduce the use of the calcineurin inhibitor, but the long-term use is inevitable in some patients with transplantation or steroid resistant nephrotic syndrome. Histological changes due to CsA persist or progress even after stopping the drug^21^. Therefore, the development and application of drugs inhibiting important mechanisms that can cause chronic CsA nephrotoxicity may be the best way to block irreversible renal histological changes such as tubulointerstitial fibrosis beforehand^28^.

In our study, we selected the MZR and LSRT for the potential drugs whether these drugs could prevent acute and chronic CsA nephrotoxicity based on previous studies^12^,^13^. MZR is extracted from the Eupenicillium brefeldianum as a purine nucleotide analog^29^, and has been used in the treatment of lupus nephritis and nephrotic syndrome^30^. Previous studies showed that MZR inhibits macrophage infiltration which has an important role in the development of interstitial fibrosis in rats with obstructive renal disease^31^,^32^.

We found that 4-week administration of CsA did not cause the development of a typical chronic CsA nephrotoxicity and rather showed the findings of acute CsA nephrotoxicity such as vacuolization and detachment from the tubular basement membrane of renal tubular cells. Although there has been no previous report on the effect of mizoribine on acute CsA nephrotoxicity, we showed that MZR had a beneficial effect on the acute renal tubular changes by CsA through decreasing renal expression of OPN and TGF-β1. However, this effect was not observed in LSRT-treated group in our study. We also demonstrated that MZR and LSRT had protective effects on inflammatory process in chronic CsA nephropathy and led to improvement of tubular damage, tubulointerstitial fibrosis and arteriolopathy by down regulation of OPN and TGF-β1, which were similar to a few previous studies.

However, previous researches have not investigated whether the MZR or LSRT might have a beneficial effect on glomerular growth^12^,^13^, which could be decreased by CsA. We thought that measurement of glomerular growth will be important because long-term use of CsA is also inevitable in some children as well as in adults and a younger age at the start of CsA is known to be one of the important factor for the development of chronic CsA nephrotoxicity in children with nephrotic syndrome^8^. It was reported that the glomerular growth in rats increases linearly from the age of five weeks to two years with increasing age^33^. A previous report showed that long-term administration of CsA resulted in a reduction in glomerular volume, especially volume of the glomerular vascular ring in rats^34^ and the impact of CsA on glomerular volume has also been reported both in adults and children, including our previous study^35^,^36^. Because glomerular growth is more active during childhood than during adulthood, we thought that the effect of CsA might be greater in children than in adults and effective inhibition of renal toxicity with the MZR or LSRT could also affect glomerular growth.

We firstly demonstrated that glomerular area significantly decreased in the CsA group than that in control group after 4-week and 7-week administration of CsA, which were reversed by coadministration of MZR and LSRT. Glomerular area was measured with both the Bowman’s capsule and glomerular vascular ring area and both glomerular areas were decreased, but the reduction of Bowman’s capsule area was more prominent in the CsA group. Both glomerular areas were improved and Bowman’s capsule area was well-maintained after MZR or LSRT treatment. These results suggest that there has been the collapse of the Bowman’s space and glomerular vascular ring area through progression of interstitial fibrosis by CsA, which was improved by MZR or LSRT.

Regarding the dose of losartan, the rats showed growth failure and a reduced survival due to intolerability of LSRT (100 mg/L) in the first experiment, but the rats showed good tolerance and growth to the adjusted dose of LSRT (35 mg/L) in the second experiment. Because co-administration of LSRT (35 mg/L) with CsA demonstrated the inhibitory effect for chronic nephrotoxicity and impairment of glomerular growth by CsA, it is thought to require a consideration for the tolerability of LSRT by dose adjustment.

In conclusion, our study demonstrated that MZR and LSRT had protective effects on inflammatory process in acute and chronic CsA nephropathy and led to improvement of tubular damage, tubulointerstitial fibrosis and arteriolopathy by down regulation of OPN and TGF-β1 and glomerular size contraction. Given these results, we speculate that MZR and LSRT can be used alone or in combinations as the potential antagonists for the inhibition of CsA nephrotoxicity. Further studies are necessary to evaluate the combined use of these drugs with CsA could prevent chronic CsA nephrotoxicity and glomerular area contraction in nephrotic children who are receiving long-term CsA treatment.

## Materials and Methods

### Experimental animals and groups

The 6-week-old male Sprague-Dawley rats were bred under specific-pathogen-free (SPF) conditions and low salt diet (0.05%). At the first experiment, they were divided into eight experimental groups and CsA were administered for 4 weeks. Then they were sacrificed and kidney biopsies were performed to investigate the expression of acute renal toxicity. At the second experiment, CsA were administered for 7 weeks and investigated chronic CsA nephrotoxicity.

- The control group (VH): CsA vehicle (olive oil) group
- VH + Losartan (LSRT) group
- VH + Mizoribine (MZR) group
- CsA group
- CsA + LSRT group
- CsA + MZR group
- CsA + LSRT + MZR group

### Experimental drugs

#### A. Cyclosporine A (CsA) (Novartis Pharma Co., USA)

CsA was dissolved in the olive oil (Sigma Co., St. Louis, MO) as a vehicle and a final concentration of 15 mg/mL was made, which was administered subcutaneously with the dose of 15 mg/kg/day.

#### B. Losartan (LSRT)

LSRT was dissolved in purified water at a concentration of 100 mg/L and mixed with the drinking water and then administered (target volume; 30 mg/kg/day) in the first experiment during 4 weeks, and at the second experiment the dose of LSRT reduced to 35 mg/L during 6 weeks because of intolerability showed in the first experiment.

#### C. Mizoribine (MZR) (Bredinin: Asahi Chemical Industry Co., Japan)

MZR was dissolved in normal saline to make a final concentration of 5 mg/mL and administered with the dose of 5 mg/kg/day by intraperitoneal injection.

### Histological CsA nephrotoxicity and semi-quantitative scoring

We checked the CsA nephrotoxicity 4 and 7 weeks after CsA administration using PAS and trichrome stain by light microscopy in each group. Histological nephrotoxicity was classified as non-specific acute tubular changes and characteristic chronic nephrotoxicity according to the tissue changes caused by CsA using a semi-quantitative scoring system in each group.

#### A. Acute tubular changes

They were semiquantitatively scored as 1–4 points according to the proportion of tubular cells which showed vacuolization and detachment from the tubular basement membrane (1 point: none, 2 points: <25%, 3 points: 25–50% and 4 points: >50%).

#### B. Tubulointerstitial changes due to chronic renal toxicity

They were semiquantitatively scored as 1–4 points according to the proportion of tubular cells showed tubular atrophy, interstitial fibrosis and mononuclear infiltration (1 point: none, 2 points: <25%, 3 points: 25–50%, 4 points: >50%).

#### C. Arteriolar lesions caused by chronic renal toxicity (arteriolopathy)

They were semiquantitatively scored as 1–4 points according to the proportion and type of arteriolar changes (1 point: none, 2 points: <50% and non-circumferential, 3 points: >50% frequent and non-circumferential and 4 points: circumferential).

### Measurement of glomerular area

Renal tissue was PAS stained and magnified (×400) by light microscope. The image was captured and switched to JPEG file. Using JPEG image files converted by using a computer program Image Pro Plus, the glomeruli which have the pole of the glomerular vasculature were selected and the Bowman’s capsule and glomerular vascular ring were traced and 15 glomerular area in each experiment object was measured.

### Real-time reverse transcription polymerase chain reaction (RT-PCR)

The expression levels of OPN and TGF-β1 were measured by real-time RT-PCR using Taqman probe which has a fluorescent dye that is only at the end of 5′. Glyceraldehydes-3-phosphate dehydrogenase (GAPDH) was used as a control. Amplification and detection equipment was LightCycler480 (Roche, Germany) and PCR reaction profile was as follows (50 °C 2 minutes, 95 °C 15 minutes, 1 cycle, 95 °C 15 seconds, 60 °C 1 minute 40 cycles).

### Immunohistochemistry

The paraffin embedded tissue was dissected as 4 μm in thickness for immunohistochemical staining and paraffin was removed by xylene. It was processed with hydration in a high concentration to a low concentration of alcohol and then washed with distilled water and put it in citrate buffer (pH 6.0) and was treated twice for 5 minutes in a microwave oven. After cooling at room temperature, it was washed for 10 min in Tris buffer and for 10 minutes in 0.3% hydrogen peroxide, thereby inhibiting the endogenous peroxidase activity. After washing for 10 minutes in Tris buffer and for 15 minutes in normal blocking solution, it was reacted with the primary antibody for OPN (Abcam, Cambridge, UK, 1: 200), TGF-β1 (Serotec, Oxford, UK, 1: 500), anti-rat ED- 1 (CD68) (Serotec, Oxford, UK, 1: 200).

Using LSAB kit (DAKO, Denmark), it was sequentially reacted with secondary antibody coupled to biotin and streptavidin-biotin peroxidase. It was stained with the diaminobenzidine (DAB) and then counterstained with Harris hematoxyline, and the differences in expression in control and treated animals were analyzed.

We counted ED-1 (+) cells per high power field on microscopy which were represented as the average by counting the area of the 3 different areas.

### Blood chemistry tests

After administration of experimental drugs, blood chemistry tests (blood urea nitrogen [BUN], creatinine, cholesterol, triglyceride) and blood CsA concentrations were measured. Whole blood CsA concentrations were measured by monoclonal radioimmunoassay. CsA levels and blood chemistry tests were performed after last administration of CsA and just before sacrifice of rats. In untreated controls, sampling data before sacrifice were used and therefore, we did not perform blood sampling in all groups prior to drug administration.

### Statistical analysis

The data was analyzed to compare the mean differences between the experimental and control group by an independent t-test and ANOVA using SPSS for Windows, version 18.0 (SPSS Inc., Chicago, Illinois, USA). All differences were considered significant at a value of P < 0.05.

## Additional Information

**How to cite this article**: Kim, J. H. *et al.* Influence of cyclosporine A on glomerular growth and the effect of mizoribine and losartan on cyclosporine nephrotoxicity in young rats. *Sci. Rep.* **6**, 22374; doi: 10.1038/srep22374 (2016).

## Supplementary Material

Supplementary Information

## Figures and Tables

**Table 1:** Acute tubular changes in rat kidney after treatment of CsA for 4 weeks.

Treatment groups	N	Vacuolization and drop out of tubular epithelial cell	No. of positive cases	P value
VH^a^	10	Absent to mild	10	< 0.01^a^
		Moderate to severe	0	
CsA^a,b^	15	Absent to mild	4	
		Moderate to severe	10	
CsA + L(50mg/L)	3	Absent to mild	2	
		Moderate to severe	1	
CsA + L(100mg/L)	4	Absent to mild	4	
		Moderate to severe	0	
CsA + M^b^	12	Absent to mild	9	< 0.01^b^
		Moderate to severe	3	
CsA + L(50mg/L) + M	4	Absent to mild	4	
		Moderate to severe	0	
CsA + L(100mg/L) + M	2	Absent to mild	2	
		Moderate to severe	0	

N; number of subjects, VH; vehicle (olive oil), M; mizoribine, L; losartan, CsA; cyclosporine A, Score of acute CsA toxicity (% of vacuolization and drop out of tubular epithelial cell); 0: none, 1: <25%, 2: 25–50%, 3, >50%.

**Table 2:** Glomerular area and kidney weight after treatment of CsA for 4 weeks.

Treatment groups	N	Glomerular Bowman’s area (10^5^μm^2^)	Glomerular tuft area (10^5^μm^2^)	Kidney weight (g)
VH	10	12.99 ± 1.37^a^	10.72 ± 1.59^a^	1.43 ± 0.10*
CsA	15	8.51 ± 1.18a,b,c,d	6.67 ± 1.51a,b,c,d	1.03 ± 0.09*
CsA + L(50mg/L)	3	12.30 ± 1.19^b^	10.29 ± 0.70^b^	1.25 ± 0.17*
CsA + M	12	11.06 ± 1.22^c^	8.29 ± 1.06^c^	1.11 ± 0.14*
CsA + L(50mg/L) + M	4	11.62 ± 1.59^d^	10.56 ± 2.64^d^	1.07 ± 0.12*

N; number of subjects, VH; Vehicle (olive oil), M; Mizoribine, L; Losartan, CsA; cyclosporine A, Data are expressed as mean ± SD

^a,b,c,d^p < 0.01 (glomerular Bowman’s and tuft area), *p < 0.05 (kidney weight, all groups vs. VH).

**Table 3:** Tubulointerstitial changes due to chronic CsA nephrotoxicity after CsA treatment for 7 weeks.

Treatment groups	N	Histologic changes	No. ofpositivecases	P value	
Tubular atrophy					
VH^a^	6	Absent to mild	6	< 0.01^a^	
		Moderate to severe	0		
CsA^a^	8	Absent to mild	5		
		Moderate to severe	3		
CsA + M	6	Absent to mild	4		
		Moderate to severe	2		
CsA + L(35m/L) + M	4	Absent to mild	4		
		Moderate to severe	0		
Interstitial fibrosis					
VH^b^	6	Absent to mild	6	< 0.01^b^	
		Moderate to severe	0		
CsA^b,d,e^	8	Absent to mild	2		
		Moderate to severe	6		
CsA + M^d^	6	Absent to mild	6	< 0.01^d^	
		Moderate to severe	0		
CsA + L(35m/L) + M^e^	4	Absent to mild	4	< 0.05^e^	
		Moderate to severe	0		
Mononuclear cell infiltration					
VH^C^	6	Absent to mild	6	< 0.01^C^	
		Moderate to severe	0		
CsA^C^	8	Absent to mild	2		
		Moderate to severe	6		
CsA + M	6	Absent to mild	3		
		Moderate to severe	3		
CsA + L(35mgL) + M	4	Absent to mild	3		
		Moderate to severe	1		

N; number of subjects, VH; vehicle (olive oil), M; mizoribine, L; losartan, CsA; cyclosporine A, Score of chronic CsA toxicity by incidence of tubular atrophy, interstitial fibrosis, mononuclear cell infiltration (0: none, 1: <25%, 2: 25–50%, 3, >50%).

**Table 4:** Arteriolopathy after CsA treatment for 7 weeks.

Treatment groups	N	Grade of arteriolopathy due to CsA nephrotoxicity	No. of positive cases	P value
VH^a^	6	Absent to mild	6	< 0.01^a^
		Moderate to severe	0	
CsA^a,^*	8	Absent to mild	0	
		Moderate to severe	8	
CsA + M	6	Absent to mild	0	
		Moderate to severe	6	
CsA + L(100mg/L) + M*	4	Absent to mild	2	< 0.05*
		Moderate to severe	2	

N; number of subjects, VH; vehicle (olive oil), M; mizoribine, L; losartan.

CsA; cyclosporine A, Score of arteriolopathy due to chronic CsA toxicity (0: none, 1: rare and non-circumferential, 2: frequent and non-circumferential, 3: circumferential).

**Table 5:** Glomerular areas and kidney weight after treatment of CsA for 7 weeks.

Treatment groups	N	Glomerular Bowman’s area (10^5^μm^2^)	Glomerular tuft area (10^5^μm^2^)	Kidney weight (g) (Mean ± SD)
VH	6	13.86 ± 1.29^a^	11.37 ± 0.99^a^	1.65 ± 0.02*
CsA	8	8.54 ± 1.81a,b,c	7.75 ± 0.87a,b,c	1.10 ± 0.20*
CsA + M	8	12.82 ± 1.66^b^	10.60 ± 1.57^b^	1.06 ± 0.12
CsA + L(35mg) + M	4	12.42 ± 1.93^c^	9.75 ± 1.91^c^	1.43 ± 0.01

N; number of subjects, VH; vehicle (olive oil), M; mizoribine, L; losartan,

CsA; cyclosporine A, Data are expressed as mean ± SD

^a,b,c^p < 0.01 (glomerular Bowman’s and tuft area), *p < 0.05 (kidney weight).

## References

[b1] Klein M., Radhakrishnan J. & Appel G. Cyclosporine treatment of glomerular diseases. Annu. Rev. Med 50, 1–15 (1999). 10073260 10.1146/annurev.med.50.1.1

[b2] Cohen D. J. *et al.* Cyclosporine: a new immunosuppressive agent for organ transplantation. Ann. Intern. Med 101, 667–682 (1984). 6385799 10.7326/0003-4819-101-5-667

[b3] Faul C. *et al.* The actin cytoskeleton of kidney podocytes is a direct target of the antiproteinuric effect of cyclosporine A. Nat. Med. 14, 931–938 (2008). 18724379 10.1038/nm.1857 PMC4109287

[b4] Naesens M., Kuypers D. R. & Sarwal M. Calcineurin inhibitor nephrotoxicity. Clin. J. Am. Soc. Nephrol. 4, 481–508 (2009). 19218475 10.2215/CJN.04800908

[b5] Liptak P. & Ivanyi B. Primer: Histopathology of calcineurin-inhibitor toxicity in renal allografts. Nat. Clin. Pract. Nephrol. 2, 398–404 (2006). 16932468 10.1038/ncpneph0225

[b6] Niaudet P., Broyer M. & Habib R. Treatment of idiopathic nephrotic syndrome with cyclosporine A in children. Clin. Nephrol. 35 (Supp 1), S31–36 (1991). 1860265

[b7] Iijima K. *et al.* Risk factors for cyclosporine induced tubulointerstitial lesions in children with minimal change nephrotic syndrome. Kidney Int. 61, 1801–1805 (2002). 11967030 10.1046/j.1523-1755.2002.00303.x

[b8] Fujinaga S. *et al.* Independent risk factors for chronic cyclosporine induced nephropathy in children with nephrotic syndrome. Arch. Dis. Child. 91, 666–670 (2006). 16670120 10.1136/adc.2005.080960 PMC2083035

[b9] Kengne-Wafo S. *et al.* Risk factors for cyclosporine A nephrotoxicity in children with steroid-dependant nephrotic syndrome. Clin. J. Am. Soc. Nephrol. 4, 1409–1416 (2009). 19628686 10.2215/CJN.01520209 PMC2736699

[b10] Kim J. H. *et al.* Predictive factors for ciclosporin-associated nephrotoxicity in children with minimal change nephrotic syndrome. J. Clin. Pathol. 64, 516–519 (2011). 21441261 10.1136/jclinpath-2011-200005

[b11] Yang C. W. *et al.* Cyclosporine withdrawal and mycophenolate mofetil treatment effects on the progression of chronic cyclosporine nephrotoxicity. Kidney Int. 62, 20–30 (2002). 12081560 10.1046/j.1523-1755.2002.00400.x

[b12] Hara S. *et al.* Protective effects of Mizoribine on Cyclosporine A nephropathy in rats. Pediatr. Res. 66, 524–527 (2009). 19668109 10.1203/PDR.0b013e3181b9b48a

[b13] Endo A. *et al.* Synergistic protective effects of mizoribine and angiotensin II receptor blockade on cyclosporine A nephropathy in rats. Pediatr. Res. 75, 38–44 (2014). 24121426 10.1038/pr.2013.169

[b14] Wissmann C. *et al.* Acute cyclosporine induced nephrotoxicity in renal transplant recipients: the role of the transplanted kidney. J. Am. Soc. Nephrol. 7, 2677–2681 (1996). 8989748 10.1681/ASN.V7122677

[b15] English J. *et al.* Cyclosporine-induced acute renal dysfunction in the rat. Evidence of arteriolar vasoconstriction with preservation of tubular function. Transplantation 44, 135–141 (1987). 3603674 10.1097/00007890-198707000-00027

[b16] Scherrer U. *et al.* Cyclosporine induced sympathetic activation and hypertension after heart transplantation. N. Engl. J. Med. 323, 693–699 (1990). 2388667 10.1056/NEJM199009133231101

[b17] Pichler R. H. *et al.* Pathogenesis of cyclosporine nephropathy: roles of angiotensin II and osteopontin. J. Am. Soc. Nephrol. 6, 1186–1196 (1995). 8589285 10.1681/ASN.V641186

[b18] Kon V. *et al.* Role of endothelin in cyclosporine-induced glomerular dysfunction. Kidney Int. 37, 1487–1491 (1990). 2194067 10.1038/ki.1990.139

[b19] Bobadilla N. A. *et al.* Role of NO in cyclosporine nephrotoxicity: effects of chronic NO inhibition and NO synthases gene expression. Am. J. Physiol. 274, F791–798 (1998). 9575905 10.1152/ajprenal.1998.274.4.F791

[b20] Andoh T. F. & Bennett W. M. Chronic Cyclosporine nephrotoxicity. Curr. Opin. Nephrol. Hypertens. 7, 265–270 (1998). 9617556 10.1097/00041552-199805000-00005

[b21] Myers B. D. *et al.* Cyclosporine-associated chronic nephropathy. N. Engl. J. Med. 311, 699–705 (1984). 6382005 10.1056/NEJM198409133111103

[b22] Yang C. W. *et al.* Expression of apoptosis related genes in chronic cyclosporine nephrotoxicity in mice. Am. J. Transplant. 2, 391–399 (2002). 12123203 10.1034/j.1600-6143.2002.20501.x

[b23] Thomas S. E. *et al.* Accelerated apoptosis characterizes cyclosporine-associated interstitial fibrosis. Kidney Int. 53, 897–908 (1998). 9551396 10.1111/j.1523-1755.1998.00835.x

[b24] Pichler R. H. *et al.* Pathogenesis of cyclosporine nephropathy: roles of angiotensin II and osteopontin. J. Am. Soc. Nephrol. 6, 1186–1196 (1995). 8589285 10.1681/ASN.V641186

[b25] Shihab F. S. *et al.* Role of transforming growth factor-beta 1 in experimental chronic cyclosporine nephropathy. Kidney Int. 49, 1141–1151 (1996). 8691736 10.1038/ki.1996.165

[b26] Shihab F. S. *et al.* Sodium depletion enhances fibrosis and the expression of TGF-beta1 and matrix proteins in experimental chronic cyclosporine nephropathy. Am. J. Kidney Dis. 30, 71–81 (1997). 9214404 10.1016/s0272-6386(97)90567-9

[b27] Shihab F. S. *et al.* Pirfenidone treatment decreases transforming growth factor-beta1 and matrix proteins and ameliorates fibrosis in chronic cyclosporine nephrotoxicity. Am. J. Transplant. 2, 111–119 (2002). 12099512 10.1034/j.1600-6143.2002.020201.x

[b28] Yoon H. E. & Yang C. W. Established and newly proposed mechanisms of chronic cyclosporine nephropathy. Korean J. Intern. Med. 24, 81–92 (2009). 19543484 10.3904/kjim.2009.24.2.81 PMC2698583

[b29] Mizuno K. *et al.* Studies on bredinin. I. Isolation, characterization and biological properties. J. Antibiot. (Tokyo) 27, 775–782 (1974). 4616933 10.7164/antibiotics.27.775

[b30] Kawasaki Y. Mizoribine: a new approach in the treatment of renal disease. Clin. Dev. Immunol. 2009, 681482 (2009). 20052390 10.1155/2009/681482 PMC2801010

[b31] Sakai T., Kawamura T. & Shirasawa T. Mizoribine improves renal tubulointerstitial fibrosis in unilateral ureteral obstruction (UUO)-treated rat by inhibiting the infiltration of macrophages and the expression of alpha-smooth muscle actin. J. Urol. 158, 2316–2322 (1997). 9366383 10.1016/s0022-5347(01)68242-9

[b32] Sato N. *et al.* Mizoribine ameliorates the tubulointerstitial fibrosis of obstructive nephropathy. Nephron 89, 177–185 (2001). 11549900 10.1159/000046065

[b33] Cortes P. *et al.* Age-related changes in glomerular volume and hydroxyproline content in rat and human. J. Am. Soc. Nephrol. 2, 1716–1725 (1992). 1498277 10.1681/ASN.V2121716

[b34] Perico N. *et al.* Morphometrical analysis of glomerular changes induced by cyclosporine in the rat. Am. J. Kidney Dis. 17, 537–543 (1991). 2024655 10.1016/s0272-6386(12)80495-1

[b35] Jeong H. J. *et al.* Glomerular growth under cyclosporine treatment in childhood nephrotic syndrome. Clin. Nephrol. 55, 289–296 (2001). 11334314

[b36] Bertani T., Ferrazzi A. & Remuzzi G. Nature and extent of glomerular injury by cyclosporine in heart transplantation patients. Kidney Int. 40, 243–250 (1991). 1942772 10.1038/ki.1991.206

