# Supplementary material for: Influence of cyclosporine A on glomerular growth and the effect of mizoribine and losartan on cyclosporine nephrotoxicity in young rats
Source: Sci Rep. 2016 Mar 7;6:22374. doi: 10.1038/srep22374 (PMC4780085; doi:10.1038/srep22374)

**Influence of cyclosporine A on glomerular growth and the effect of mizoribine and losartan on cyclosporine nephrotoxicity in young rats**

Ji Hong Kim^1,2^, Yon Hee Lee^2^, Beom Jin Lim^3^, Hyeon Joo Jeong^4^,

Pyung Kil Kim^2^, Jae Il Shin^2,4^

1. Department of Pediatrics, Gangnam Severance Hospital, Yonsei University College of Medicine, Seoul, Korea
2. Department of Pediatrics, Yonsei University College of Medicine, Seoul, Korea
3. Department of Pathology, Yonsei University College of Medicine, Seoul, Korea
4. Department of Pediatric Nephrology, Severance Children’s Hospital, Yonsei University College of Medicine, Seoul, Korea

**Supplementary tables**

**Supplementary table S1. Body weight changes after treatment of CsA for 4 weeks**

**Supplementary table S2. Blood chemistry changes after treatment of CsA for 4 weeks**

**Supplementary table S3. Expression of osteopontin (spp1) and TGF-β1 mRNA in rat kidney detected by real-time PCR after treatment of CsA for 4weeks**

**Supplementary table S4. Body weight changes after treatment of CsA for 7 weeks**

**Supplementary table S5. Blood chemistry changes after treatment of CsA for 7 weeks**

**Supplementary table S6. Expression of osteopontin (spp1) and TGF-β1 mRNA1 in rat kidney detected by real-time PCR after treatment of CsA for 7 weeks**

**Supplementary table S7. Expression of anti-rat ED-1 (+) cells on immune-histochemistry after CsA treatment for 7 weeks**

**Supplementary figures**

**Supplementary fig. S1. Immunohistochemistry stain after treatment of CsA for 4 weeks.**

**Supplementary fig. S2.** Arteriolopathy showing circumferential medial hypertrophy and hyalinosis (arrow) developed in CsA-treated group (PAS stain, x400).

**Supplementary fig. S3. Immunohistochemical stain after treatment of CsA for 7 weeks**

**Supplementary fig. S4. Expression of anti-rat ED-1 (+) cell after CsA treatment for 7 weeks.**

**Supplementary tables**

**Supplementary table S1. Body weight changes after treatment of CsA for 4 weeks**

| Treatment groups | N | 0 day | 1w | 2wks | 3wks | 4wks | Δ% |
| --- | --- | --- | --- | --- | --- | --- | --- |
| VH | 10 | 218±5 | 260±9 | 300±9 | 332±13 | 355±15 | +65 |
| VH+L(50mg/L) | 4 | 224±10 | 250±11 | 272±15 | 305±22 | 322±24 | +43 |
| VH+L(100mg/L) | 8 | 218±6 | 231±10 | 250±17 | 279±24 | 290±28 | +33 |
| VH+M | 8 | 210±8 | 251±12 | 286±13 | 321±18 | 346±19 | +64 |
| VH+L(50mg/L)+M | 4 | 219±7 | 241±11 | 251±17 | 284±22 | 303±25 | +38 |
| VH+L(100mg/L)+M | 3 | 221±5 | 242±8 | 244±8 | 280±27 | 294±37 | +33 |
| CsA | 15 | 215±11 | 241±13 | 261±13 | 283±15 | 302±15* | +40 |
| CsA+L(50mg/L) | 3 | 224±8 | 230±1 | 209±29 | 228±48 | 238±53 | +6 |
| CsA+L(100mg/L) | 4 | 220±14 | 204±4 | 191±3 | 202±15 | 206±16* | -7 |
| CsA+M | 12 | 212±12 | 244±15 | 264±17 | 283±24 | 298±25 | +40 |
| CsA+L(50mg/L)+M | 4 | 225±7 | 230±12 | 245±30 | 251±43 | 233±49 | +4 |
| CsA+L(100mg/L)+M | 2 | 206±8 | 237±4 | 254±1 | 267±10 | 264±28 | +28 |

N; number of subjects, VH; vehicle (olive oil), M; mizoribine, L; losartan,

CsA; cyclosporine A, Data are expressed as mean±SD

**Supplementary table S**2. Blood chemistry changes after treatment of CsA for 4 weeks

| Treatment groups | N | CsA level  (ng/mL) | BUN  (mg/dL) | Creatinine  (mg/dL) | Cholesterol.  (mg/dL) | Triglyceride.  (mg/dL) |
| --- | --- | --- | --- | --- | --- | --- |
| VH | 10 |  | 16.1±2.5 | 0.63±0.08 | 79.0±8.9 | 138.5±43.3 |
| VH+L(50mg/L) | 4 |  | 17.9±3.5 | 0.50±0.04 | 81.9±8.7 | 102.9±19.4 |
| VH+L(100mg/L) | 8 |  | 22.6±6.9 | 0.65±0.05 | 75.5±16.5 | 89.2±29.2 |
| VH+M | 8 |  | 14.7±1.2 | 0.63±0.05 | 77.5±12.9 | 99.0±25.9 |
| VH+L(50mg/L)+M | 4 |  | 14.5±1.6 | 0.49±0.05 | 60.0±14.3 | 98.2±48.8 |
| VH+L(100mg/L)+M | 3 |  | 18.6±13.4 | 0.57±0.06 | 78.7±12.8 | 142.5±34.4 |
| CsA | 15 | 5700±897 | 67.6±26.1* | 0.85±0.18** | 103.4±11.9 | 138.0±56.2 |
| CsA+L(50mg/L) | 3 | 4615±4264 | 136.3±76.2 | 0.44±0.19 | 119.4±16.5 | 174.8±87.24 |
| CsA+L(100mg/L) | 4 | 5245±758 | 224.7±42.1 | 3.05±1.14 | 169.1±25.2 | 75.0±18.27 |
| CsA+M | 12 | 5159±908 | 19.9±12.1* | 0.55±0.28** | 69.2±30.8 | 163.4±125.9 |
| CsA+L(50mg/L)+M | 4 | 5747±2665 | 84.0±66.2 | 0.51±0.32** | 95.8±48.4 | 77.1±75.3 |
| CsA+L(100mg/L)+M | 2 | 6306±1222 | 101.6±124.7 | 0.55±0.49 | 73.0±90.5 | 50.4±25.1 |

N; number of subjects, VH; vehicle (olive oil), M; mizoribine, L; losartan, CsA; cyclosporine A, Data are expressed as mean±SD, *p<0.01, **p<0.01

**Supplementary table S3. Expression of osteopontin (spp1) and TGF-β1 mRNA in rat kidney detected by real-time PCR after treatment of CsA for 4weeks**

| Treatment groups | N | Spp1/GAPDH  mRNA  (% ratio) | p value | TGF-β1/GAPDH  mRNA  (% ratio) | P value |
| --- | --- | --- | --- | --- | --- |
| VH | 2 | *100±7 | *<0.01 | **100±8 | **<0.01 |
| VH+L(50mg/L) | 4 | 81±9 |  | 115±44 |  |
| VH+M | 8 | 106±43 |  | 150±115 |  |
| VH+L(50mg/L)+M | 4 | 134±98 |  | 127±48 |  |
| CsA | 8 | *320±94^a,b,c^ |  | **301±149^d,e^ |  |
| CsA+L(50mg/L) | 3 | 81±43^a^ | <0.03^a^ | 150±60^d^ | <0.05^d^ |
| CsA+M | 8 | 163±6^b^ | <0.01^b^ | 271±94 |  |
| CsA+L(50mg/L)+M | 4 | 81±36^,c^ | <0.02^c^ | 150±55^e^ | <0.05^e^ |

N; number of subjects, VH; vehicle (olive oil), M; mizoribine, L; losartan,

CsA; cyclosporine A, spp1; Secreted Phosphoprotein 1, TGF; transforming growth factor, Data are

expressed as mean±SD

**Supplementary table S4**. Body weight changes after treatment of CsA for 7 weeks

| Treatment groups | N | 0 day | 2wks | 4wks | 6wks | 7wks | Δ% |
| --- | --- | --- | --- | --- | --- | --- | --- |
| VH | 6 | 222±1 | 315±5 | 383±8 | 458±17 | 474±15 | 113%* |
| CsA | 8 | 230±10 | 289±12 | 317±16 | 345±14 | 368±17 | 60%* |
| CsA+M | 8 | 217±8 | 275±12 | 297±11 | 296±16 | 284±25 | 30%* |
| CsA+L(35mg/L)+M | 4 | 221±3 | 247±23 | 307±16 | 347±5 | 345±4 | 56%* |

N; number of subjects, VH; vehicle (olive oil), M; mizoribine, L; losartan,

CsA; Cyclosporine A, Data are expressed as mean±SD, *p<0.05 (all groups vs. VH)

**Supplementary table S5. Blood chemistry changes** after treatment of CsA for 7 weeks

| Treatment groups | N | Serum CsA  level (ng/mL) (Mean±SD) | BUN | Creatinine | Cholesterol. | Triglyceride |
| --- | --- | --- | --- | --- | --- | --- |
|  |  |  | (mg/dL)  (Mean±SD) | (mg/dL) (Mean±SD) | (mg/dL)  (Mean±SD) | (mg/dL)  (Mean±SD) |
| VH | 6 |  | 20.7±1.9 | 0.7±0.1 | 101.9±24.2 | 98.7±12.4 |
| CsA | 8 | 4479±963 | 89.7±32.2 | 1.1±0.3 | 98.0±11.9 | 125.1±32.9 |
| CsA+M | 8 | 3920±1102 | 101.7±26.6 | 1.17±0.2 | 101.7±5.5 | 178.4±9.8 |
| CsA+L(35mg)+M | 4 | 4907±1805 | 73.5±1.6 | 0.6±0.1 | 113.2±3.9 | 138.0±26.2 |

N; number of subjects, VH; vehicle (olive oil), M; mizoribine, L; losartan,

CsA; cyclosporine A, BUN, blood urea nitrogen, Data are expressed as mean±SD

**Supplementary table S6. Expression of osteopontin (spp1) and TGF-β1 mRNA1 in rat kidney detected by real-time PCR after treatment of CsA for 7 weeks**

| Treatment groups | N | Spp1/GAPDH mRNA  (% ratio) | P value | TGF-β1/GAPDH mRNA  (% ratio) | P value |
| --- | --- | --- | --- | --- | --- |
| VH | 6 | 100±109^a^ | <0.01^a^ | 100±33^a^ | <0.01^a^ |
| CsA | 8 | 1411±686^a,b^ |  | 355±70^a,b,c^ |  |
| CsA+M | 6 | 1383±493 |  | 165±24^b^ | <0.01^b^ |
| CsA+L(35mg)+M | 4 | 674±155^b^ | <0.05^b^ | 241±43^c^ | <0.01^c^ |

N; number of subjects, VH; vehicle (olive oil), M; mizoribine, L; losartan, CsA; cyclosporine A, TGF;

transforming growth factor, Data are expressed as mean±SD

**Supplementary table S7. Expression of anti-rat ED-1 (+) cells on immune-histochemistry after CsA treatment for 7 weeks**

| Treatment  groups | N | ED-1(+) cell count  (Mean±SD) | P value |
| --- | --- | --- | --- |
| VH | 6 | 1.7±0.1^a^ | <0.01^a^ |
| CsA | 8 | 63.6±3.8^a,b,c^ |  |
| CsA+M | 6 | 34.1±5.4^b^ | <0.01^b^ |
| CsA+L(35mg)+M | 4 | 18.3±1.4^c^ | <0.01^c^ |

N; number of subjects, VH; vehicle (olive oil), M; mizoribine, L; losartan, CsA; cyclosporine A, endothelin; ED

**Supplementary figures**

**Supplementary fig. S1. Immunohistochemistry stain after treatment of CsA for 4 weeks.**

**
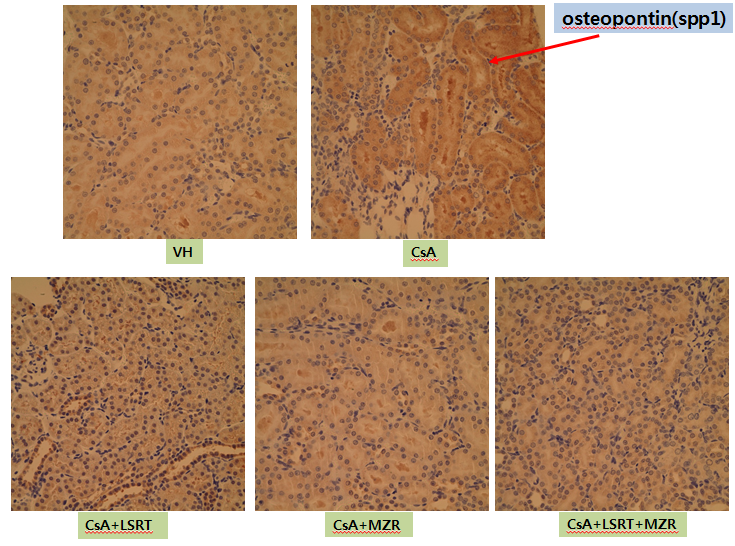
**

**
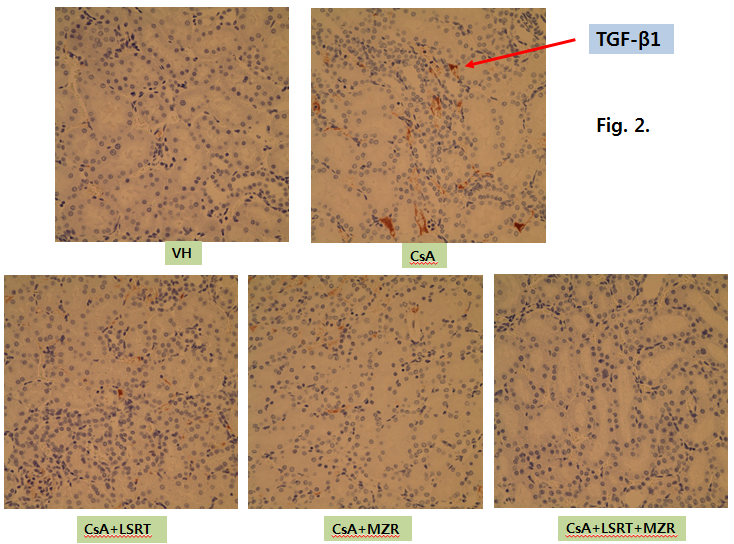
**

**Supplementary fig. S2. Arteriolopathy showing circumferential medial hypertrophy and hyalinosis (arrow) developed in CsA-treated group (PAS stain, x400).**


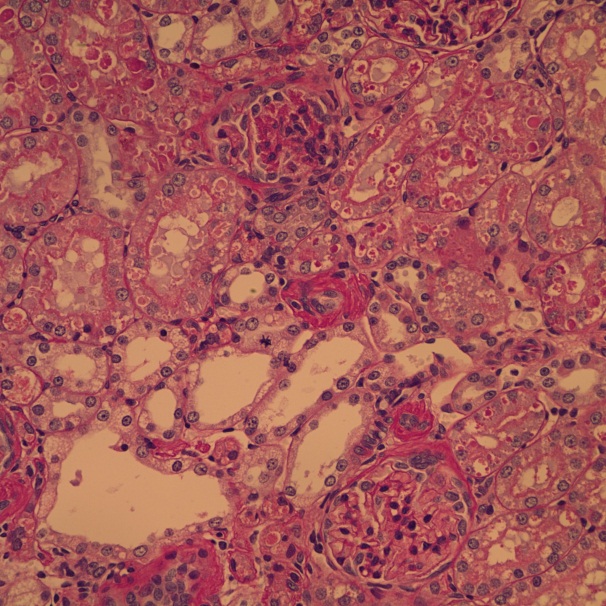


**Supplementary fig. S3. Immunohistochemical stain after treatment of CsA for 7 weeks**

**
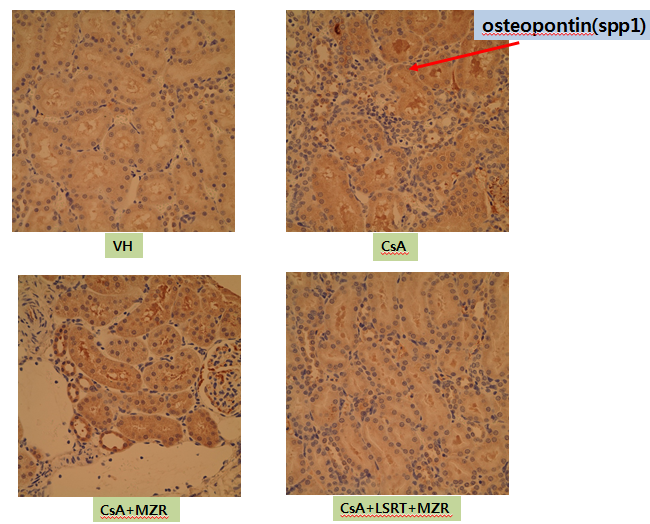
**

**
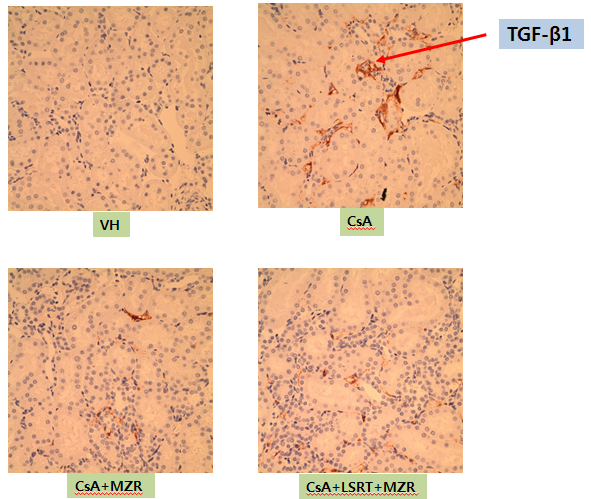
**

**Supplementary fig. S4. Expression of anti-rat ED-1 (+) cell after CsA treatment for 7 weeks.**

CsA-treated group showed significant increase of anti-rat ED-1(+) cell and mizoribine, mizoribine+losartan-treated group showed significant reduced anti-rat ED-1 (+) cells than CsA-treated group.


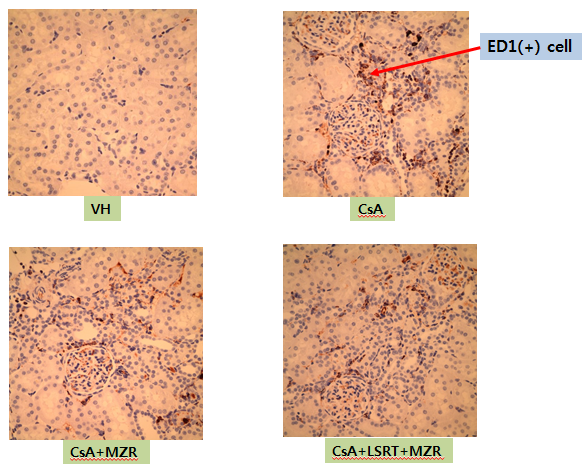

Supplement: Supplementary Information [file srep22374-s1.doc]
